# Pediatric chronic kidney disease mortality in Brazil—A time trend analysis

**DOI:** 10.1371/journal.pgph.0002304

**Published:** 2024-01-24

**Authors:** Arnauld Kaufman, André L. Barreira, Marcelo G. P. Land

**Affiliations:** 1 Pediatric Nephrology Service, Childcare and Pediatrics Institute Martagão Gesteira and Clinical Medicine Graduate Medical Program, Federal University of Rio de Janeiro, Rio de Janeiro, Brazil; 2 Nephrology Service, Clementino Fraga Filho University Hospital, Federal University of Rio de Janeiro, Rio de Janeiro, Brazil; 3 Pediatric Department, Medical School and Clinical Medicine Graduate Medical Program, Federal University of Rio de Janeiro, Rio de Janeiro, Brazil; Institute for Epidemiology, Biostatistics and Prevention, University of Zurich, SWITZERLAND

## Abstract

Chronic kidney disease (CKD) is defined based on structural or functional abnormalities of the kidneys, or a glomerular filtration rate (GFR) below the threshold of 60 ml/min per 1.73 m^2^ for more than 3 months. It is an important noncommunicable disease with a rising worldwide, becoming a global public health problem. There are few studies about this problem, especially in low- and middle-income countries (LMIC), including Brazil, an upper-middle-income country. The objective of the study was to determine the cause-specific mortality rates for pediatric CKD patients (CKDMR) from 0 to 19 years old, based on the 10^th^ revision of the International Classification of Diseases (ICD-10) and the Global Burden of Diseases Injuries and Risk Factors Study’s (GBD) list. We calculated the impact of the annual human development indexes (HDI) in CKDMR in Brazil and its regions at two different times and compared it with the literature results. We obtained data from the Department of Informatics of the Brazilian Unified Health System (DATASUS) from 1996 to 2017. The Joinpoint regression analyses estimated the average annual percentage changes (AAPCs). The correlation between the HDI values and the number of deaths from each age group in Brazil and its different regions were assessed using the time series autoregressive integrated moving average (ARIMA) models. There were 8838 deaths in a pediatric and adolescent population of about 1.485 x 10^9^ person-years observed in Brazil from 1996 to 2017. Our results demonstrated a significant increase in the AAPC in Brazil’s less than 1-year-old age group and a decrease in children from 5 to 19 years old. We observed a positive correlation between CKDMR and HDI among children under 1 year of age. Conversely, there is a negative association in the age groups ranging from 5 to 19 years, indicating an inverse relationship between CKDMR and HDI.

## 1. Introduction

Chronic kidney disease (CKD) is a progressive and irreversible disorder that reduces life quality and expectancy at all ages [1, 2]. The prevalence of CKD is rising worldwide, becoming a public health problem predominantly in low- and middle-income countries (LMIC) [1, 3–5]. Clinical trials, outcome studies, and registries in LMICs with a predominant focus on children and adolescents are limited, as indicated by previous research [6–8].

In LMICs, there is a growth in non-communicable diseases as a result of urbanization and a demographic transition, which refers to the shift in vital rates from high to low fertility rates and mortality, with a change in mortality rates caused by chronic and non-communicable diseases’ predominance [9, 10]. These demographic and disease transitions have produced an increase in kidney disease [6],

The Global Burden of Diseases, Injuries, and Risk Factors studies have estimated the mortality rates in children from 5 to 14 years of age from 1990 to 2016, in 51 countries in the WHO European Region, with substantial diversity in terms of socioeconomic and health risks between the countries [11].

In Brazil, regional variations in the demographic transition are evident, with the Southeast (SE) experiencing a faster transition marked by the stabilization of mortality rates. This stands in contrast to the North (N) and Northeast (NE) regions, characterized by slower demographic transitions and lower socioeconomic development, where a notable decline in mortality rates occurs. This observation underscores the diverse pace and dynamics of demographic shifts across different areas of the country. [12]. The distribution of professionals and resources in the Brazilian health system is concentrated in the large metropolitan regions, predominantly located in the SE region [13]. Other Brazilian regions are the South (S) and the Central-West (CW).

This study aimed to report the prevalence of pediatric CKD mortality in Brazil, using data from the Department of Informatics of the Brazilian Unified Health System (DATASUS), from 1996 to 2017. We studied the CKD mortality in 5 pediatric age groups between all Brazilian regions and compared it with the human development index (HDI) of the country. Furthermore, we compared our results with the European study data on CKD mortality rates kindly provided by the authors [11], with Brazilian data and its regions and correlated these data with the HDI of Brazil and the European countries.

## 2. Methods

### 2.1 Definitions

CKD is defined based on the presence of structural or functional abnormalities of the kidneys, or a glomerular filtration rate (GFR) below the threshold of 60 ml/min per 1.73 m^2^ for more than 3 months [14].

### 2.2. Data sources

Brazil is the largest country in South America and the 5^th^ in the world, with more than 207 million people in 2017, and a population density of 24.40 persons/km^2^. The country is geopolitically divided into 5 macro-regions presented in Table 1 [12, 15].

**Table 1:** Macro-regions of Brazil.

Brazilian region	Percentual of the Brazilian area (%)	Demographic density (persons/Km^2^)
North (N)	45.27	4.66
Northeast (NE)	18.30	36.89
Central-West (CW)	18.86	9.88
Southeast (SE)	10.85	94.04
South (S)	6.75	51.40

CKDMR was estimated based on the 10^th^ revision of the International Classification of Diseases [ICD-10], using the Global Burden of Diseases, Injuries, and Risk Factors Study for causes of death diagnostic codes for CKD, as the same as the European study, listed in Table 2 [11, 16]. From August to December 2019, we obtained aggregated mortality data from the federative units in Brazil from the DATASUS public universal access database. These data were sourced from the database of the DATASUS Mortality Information System (Sistema de *informação de mortalidade–SIM*) and covered the period from 1996 to 2017 [17].

**Table 2:** ICD-10 version 2015.

Code	Disease
D63	Anemia in chronic diseases classified elsewhere
E10.2	Type 1 diabetes mellitus with kidney complications
E11.2	Type 2 diabetes mellitus with kidney complications
E13.2	Other specified diabetes mellitus with kidney complications
E14.2	Unspecified diabetes mellitus with kidney complications.
I12	Hypertensive kidney disease
I13	Hypertensive heart and kidney disease
N02	Recurrent and persistent hematuria
N03	Chronic nephritic syndrome
N04	Nephrotic syndrome
N05	Unspecified nephritic syndrome
N06	Isolated proteinuria with specified morphological lesion
N07	Hereditary nephropathy, not elsewhere classified
N08	Glomerular disorders in diseases classified elsewhere
N15	Other tubulointerstitial kidney diseases
N18	Chronic kidney disease
Q61	Cystic kidney disease
Q62	Congenital obstructive defects of renal pelvis and congenital malformations of ureter

Other variables studied were the annual population of Brazil and its regions, sex, and age divided into the age groups of children less than 1 year, 1 to 4 years, 5 to 9 years, 10 to 14 years, and 15 to 19 years, and the HDI for Brazil, published annually and extracted from the Human Development Indices and Indicators: 2018 Statistical Update [18] website.

### 2.2 Statistical analysis

All the formulas used in this study are presented in the supplementary material.

#### 2.2.1. Time trend analysis

CKDMR was calculated for each age group (less than 1 year, 1 to 4, 5 to 9, 10 to 14, and 15 to 19) for Brazil as a whole and its different regions and expressed per 100,000 children per year. The annual percentage changes (APC) and the average annual percentage changes (AAPC) of each CKDMR were estimated using the Poisson variance in the corrected error option, while the presence of possible breaks in the temporal trends was assessed with the Joinpoint regression analysis. To identify changes in mortality rate trends, Joinpoint regression was estimated for every age and sex group by using the Joinpoint Regression Program, Version V4.8.0.1 (Statistical Research and Applications Branch, National Cancer Institute). The number of join points is obtained using a permutation test via Monte Carlo resampling [19], and compared by estimating their Bayesian Information Criterion (BIC) [20].

#### 2.2.2. Time trend analysis for HDI regressor

The association between the annual HDI values in Brazil and the CKDMR in each age group in both sexes and its different regions in Brazil was assessed using the time series ARIMA models with regressor [21].

#### 2.2.3. Pairwise multiple comparison adjustment

To compare the proportion of death in the population by different regions in each age group, the chi-square test was used. To correct multiple comparisons, we use the Bonferroni procedure.

#### 2.2.4. Curve estimation to regional (countries) analysis (no spatial correlation) in the same period

To estimate the correlation between the HDI and CKDMR in the same periods (2000 and 2016) using the European Countries and Brazil, we use the CURVEFIT function of SPSS to determine the best curve model for our data [22].

Statistical analyzes were performed using the IBM SPSS Statistics 20 software and the Joinpoint regression program (V4.8.0.1, National Cancer).

## 3. Results

We observed a group of approximately 1.485 x 10^9^ pediatric and adolescent person-years in Brazil from 1996 to 2017, distributed as follows: 0.069 x 10^9^ from less than 1 year old, 0.281 x 10^9^ from 1 to 4 years, 0.363 x 10^9^ from 5 to 9 years, 0.384 x 10^9^ from 10 to 14 years, and 0.388 x 10^9^ from 15 to 19 years. The CKDMR according to the age group and years for Brazil and regions are presented in Table 3.

**Table 3:** CKDMR according to age groups and years for Brazil and its regions by joinpoint regression.

Region/age range	CKDMR	AAPC (95% CI)	p
BR < 1 year	3.4618	2.5 (1.4 to 4.3)	**0.016**
BR 1–4 years	0.3793	0.5 (-0.3 to 1.2)	0.118
BR 5–9 years	0.2562	-1.6 (-2.6 to -0.6)	**0.025**
BR 10–14 years	0.3474	-3.3 (-5.0 to -1.5)	**0.024**
BR 15–19 years	0.8030	-2.1 (-3.6 to -0.5)	**0.041**
N < 1 year	2.0193	4.0 (1.7 to 6.3)	**0.022**
N 1–4 years	0.4774	0.4 (-2.4 to 3.2)	0.292
N 5–9 years	0.3057	-1.6 (-4.7 to 1.5)	0.144
N 10–14 years	0.3746	-1.2 (-3.6 to 1.2)	0.159
N 15–19 years	0.8024	-1.4 (-2.9 to -0.0)	0.059
NE < 1 year	2.7215	4.3 (2.9 to 5.7)	**0.007**
NE 1–4 years	0.4394	1.0 (-0.6 to 2.6)	0.118
NE 5–9 years	0.3375	-0.1 (-1.9 to 1.8)	0.315
NE 10–14 years	0.4276	-1.8 (-3.3 to -0.3)	**0.044**
NE 15–19 years	0.9121	-0.4 (-1.6 to 0.8)	0.214
SE < 1 year	4.0102	1.7 (-0.2 to 3.7)	0.082
SE 1–4 years	0.3343	-0.1 (-1.5 to 1.3)	0.315
SE 5–9 years	0.2108	-3.7 (-5.1 to -2.2)	**0.012**
SE 10–14 years	0.2973	-5.9 (-11.8 to 0.3)	0.069
SE 15–19 years	0.7553	-3.1 (-4.9 to -1.2)	**0.028**
S < 1 year	4.0743	3.1 (1.2 to 5.1)	**0.025**
S 1–4 years	0.2855	1.0 (-2.2 to 4.4)	0.234
S 5–9 years	0.1742	-1.3 (-4.7 to 2.2)	0.194
S 10–14 years	0.2834	-2.8 (-5.0 to -0.4)	**0.044**
S 15–19 years	0.6507	-1.6 (-3.2 to -0.0)	0.059
CW < 1 year	4.5726	2.8 (0.6 to 5.1)	**0.041**
CW 1–4 years	0.3616	-1.4 (-4.2 to 1.5)	0.159
CW 5–9 years	0.2286	-0.4 (-3.5 to 2.8)	0.292
CW 10–14 years	0.3507	-0.7 (-3.4 to 2.1)	0.255
CW 15–19 years	0.8936	-3.0 (-4.4 to -1.5)	**0.018**

BR = Brazil; N = North; NE = Northeast; SE = Southeast; S = South; CW = Central-West; CMR = crude mortality rates; AAPC = average annual percentage changes

### 3.1. CKD mortality comparison in Brazil and regions

The CKDMR in the period from 1996 to 2017 in the age groups less than 1-year-old, 1 to 4 years, 5 to 9 years, 10 to 14 years, and 15 to 19 years related to Brazil and its regions (N, NE, SE, S, and CW) were compared by the chi-square test for two samples (Table 4).

**Table 4:** Statistical significance (p-value) for CKDMR regions comparison by age groups.

**Less than 1 year**						
	BR	N	NE	SE	S	CW
BR		**<0.0001**	**<0.0001**	**0.0001**	0.0034	**<0.0001**
N	<0.0001		0.0013	**<0.0001**	**<0.0001**	**<0.0001**
NE	<0.0001	0.0013		**<0.0001**	**<0.0001**	**<0.0001**
SE	0.0001	<0.0001	**<0.0001**		0.7921	0.0679
S	0.0034	<0.0001	**<0.0001**	0.7921		0.1651
CW	**<0.0001**	**<0.0001**	**<0.0001**	0.0679	0.1651	
**1–4 years**						
	BR	N	NE	SE	S	CW
BR		0.0103	0.0141	0.0382	0.0052	0.6765
N	0.0103		0.4026	**0.0003**	**<0.0001**	0.0450
NE	0.0141	0.4026		**0.0002**	**<0.0001**	0.1091
SE	0.0382	**0.0003**	**0.0002**		0.1528	0.5218
S	0.0052	**<0.0001**	**<0.0001**	0.1528		0.1075
CW	0.6765	0.0450	0.1091	0.5218	0.1075	
**5–9 years**						
	BR	N	NE	SE	S	CW
BR		0.0755	**<0.0001**	0.0036	**0.0006**	0.3921
N	0.0755		0.3559	**0.0007**	**<0.0001**	0.0678
NE	**<0.0001**	0.3559		**<0.0001**	**<0.0001**	0.0048
SE	0.0036	**0.0007**	**<0.0001**		0.1208	0.5674
S	**0.0006**	**<0.0001**	**<0.0001**	0.1208		0.1057
CW	0.3921	0.0678	0.0048	0.5674	0.1057	
**10–14 years**						
	BR	N	NE	SE	S	CW
BR		0.3981	**<0.0001**	0.0045	0.0189	0.9296
N	0.3981		0.1655	0.0173	0.0180	0.6191
NE	**<0.0001**	0.1655		**<0.0001**	**<0.0001**	0.0738
SE	0.0045	0.0173	**<0.0001**		0.6163	0.1420
S	0.0189	0.0180	**<0.0001**	0.6163		0.1040
CW	0.9296	0.6191	0.0738	0.1420	0.1040	
**15–19 years**						
	BR	N	NE	SE	S	CW
BR		0.9903	**0.0003**	0.0751	**0.0002**	0.1024
N	0.9903		0.0542	0.3602	0.0088	0.2128
NE	**0.0003**	0.0542		**<0.0001**	**<0.0001**	0.7693
SE	0.0751	0.3602	**<0.0001**		0.0151	0.0154
S	**0.0002**	0.0088	**<0.0001**	0.0151		**0.0001**
CW	0.1024	0.2128	0.7693	0.0154	**0.0001**	

BR = Brazil; N = North; NE = Northeast; SE = Southeast; S = South; CW = Central-West; CKDMR = CKD mortality rates.

The results of the comparison of the S, SE, and CW regions show no statistical differences with each other as well as the comparison between the N and NE regions. The S, SE, and CW regions have a higher HDI compared to the N and NE regions. These regions have a greater CKDMR but a smaller AAPC in the under-1-year age group compared to the N and NE regions. Additionally, there is a more significant reduction in AAPC in groups above 5 years old in the S, SE, and CW regions. All regions did not show any difference in the group of children aged between 1 and 4 years old. The SE and NE regions, which are the most populated in Brazil and represent the most and least developed areas, exhibited more pronounced disparities.

### 3.2. Time trends

Analyzing each age group, there is a significant increase in the AAPC from 1996 to 2017 in the less than 1 year age group in Brazil and its regions (AAPC between 4.3 and 1.7), except in the SE region where it was observed stability in the period (p = 0.082) (Table 3). The estimated annual percent changes of CKDMR from the segmented regression annual percentage changes analyses (sAPC) are presented in S1 Table.

In the 1 to 4 years range, we observed stability in the AAPC in Brazil and its regions.

For the 5 to 9 years group, we observed a decrease in the AAPC in Brazil (AAPC -1.6 95% CI -2.6 to -0.6 *p* = 0.025) and in the SE region (AAPC -3.7 95% CI -5.1 to—2.2 *p* = 0.012), remaining stable in other regions.

In the range of 10 to 14 years, we observed a decrease in AAPC in Brazil (AAPC -3.3 95% CI -5.0 to -1.5 *p* = 0.024), in the NE region (AAPC -1.8 95% CI -3.3 to—0.3 *p* = 0.044) and S region (AAPC -2.8 95% CI -5.0 to -0.4 *p* = 0.044), with a downward trend in the SE region (AAPC -5.9 95% CI -11.8 to 0.3 *p* = 0.069) and stability in other regions.

For the 15 to 19 years group, we observed a decrease in AAPC in Brazil (AAPC -2.1 95% CI -3.6 to -0.5 *p* = 0.041), in the SE region (AAPC -3.1 95% CI -4.9 to—1.2 *p* = 0.028) and the CW region (AAPC -3.0 95% CI -4.4 to -1.5 *p* = 0.018), with stability in the other regions.

**Fig 1:**
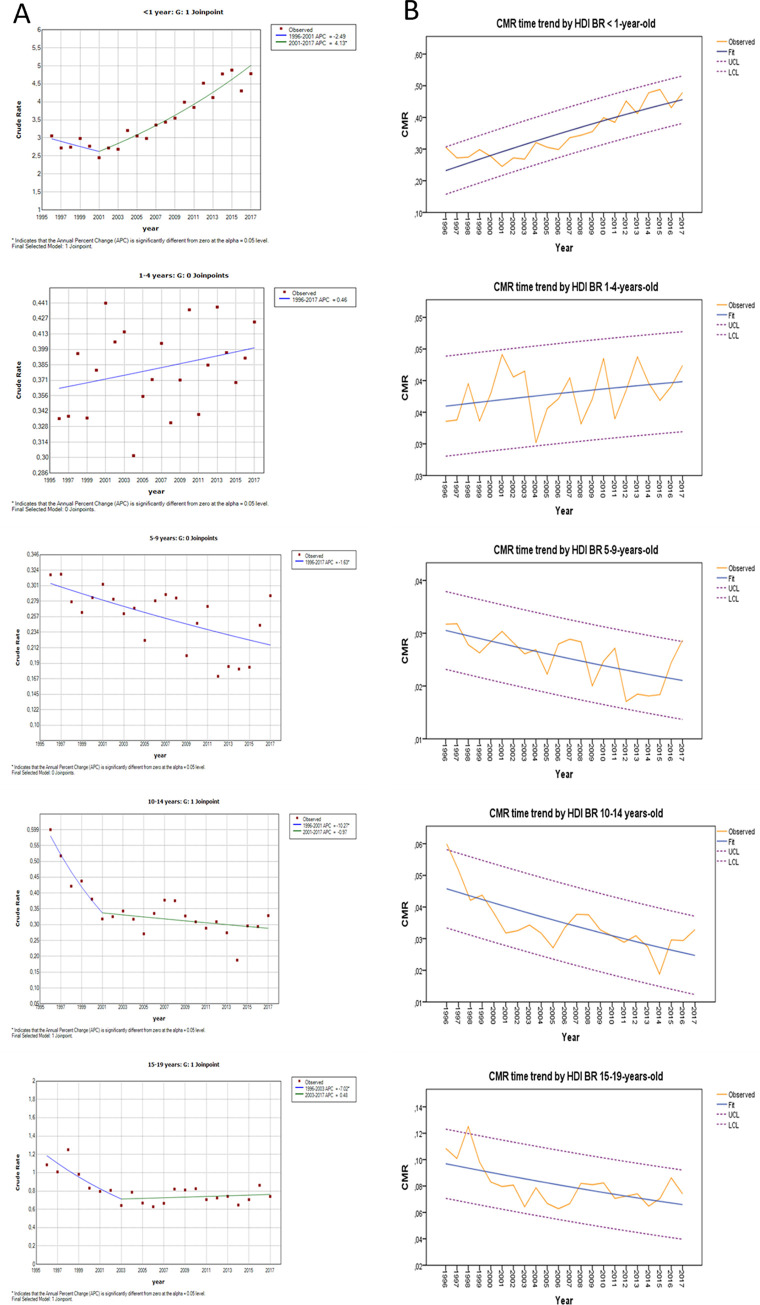
A. Joinpoint regression analysis for CKDMR APC and AAPC for Brazil and its 5 age groups. B: CKDMR time trend adjusted by HDI for Brazil and its 5 age groups.

The chart of Joinpoint regression analysis for APC and AAPC for Brazil and its 5 age groups is shown in Fig 1A.

### 3.3. CKD mortality rates adjusted by HDI (Time trend regression analysis)

Time trend regression analysis is presented in Table 5. It showed a statistically significant positive association between CKDMR in the age group of children less than 1 year in Brazil and its regions and the HDI (Brazil HDI regression coefficient 1.206, 95% CI 0.940 to 1.472 *p*<0.001; N region HDI regression coefficient 0.849, 95% CI 0.396 to 1.303 *p* = 0.002; NE region HDI regression coefficient 1.232, 95% CI 0.760 to 1.704 *p*<0.001; region SE HDI regression coefficient 1.141, 95% CI 0.593 to 1.690 *p* = 0.001; region S HDI regression coefficient 1.420, 95% CI 0.579 to 2.260 *p* = 0.003, and CW region HDI regression coefficient 1.465, 95% CI 0.418 to 2.511 *p* = 0.013).

**Table 5:** CKDMR according to age groups and years for Brazil and its region time trend regression model for HDI.

Region/age range	HDI coef (95% CI)	p
BR < 1 year	1.206 (0.940 to 1.472)	**<0.001**
BR 1–4 years	0.021 (-0.007 to 0.049)	0.162
BR 5–9 years	-0.051 (-0.077 to -0.025)	**0.001**
BR 10–14 years	-0.113 (-0.157 to -0.069)	**<0.001**
BR 15–19 years	-0.167 (-0.260 to -0.074)	**0.002**
N < 1 year	0.849 (0.396 to 1.303)	**0.002**
N 1–4 years	0.012 (-0.125 to 0.149)	0.865
N 5–9 years	-0.061 (-0.164 to 0.042)	0.261
N 10–14 years	-0.086 (-0.187 to 0.016)	0.113
N 15–19 years	-0.140 (-0.266 to -0.014)	**0.042**
NE < 1 year	1.232 (0.760 to 1.704)	**<0.001**
NE 1–4 years	0.056 (-0.014 to 0.126)	0.134
NE 5–9 years	-0.015 (-0.082 to 0.051)	0.658
NE 10–14 years	-0.088 (-0.159 to -0.018)	**0.024**
NE 15–19 years	-0.042 (-0.157 to 0.073)	0.483
SE < 1 year	1.141 (0.593 to 1.690)	**0.001**
SE 1–4 years	-0.004 (-0.055 to 0.047)	0.885
SE 5–9 years	-0.092 (-0.126 to -0.059)	**<0.001**
SE 10–14 years	-0.165 (-0.227 to -0.103)	**<0.001**
SE 15–19 years	-0.250 (-0.388 to -0.112)	**0.002**
S < 1 year	1.420 (0.579 to 2.260)	**0.003**
S 1–4 years	0.033 (-0.078 to 0.144)	0.566
S 5–9 years	-0.046 (-0.116 to 0.023)	0.207
S 10–14 years	-0.104 (0.173 to -0.036)	**0.007**
S 15–19 years	-0.132 (-0.247 to -0.017)	**0.036**
CW < 1 year	1.465 (0.418 to 2.511)	**0.013**
CW 1–4 years	-0.072 (-0.186 to 0.041)	0.225
CW 5–9 years	0.004 (-0.077 to 0.085)	0.915
CW 10–14 years	-0.006 (-0.117 to 0.105)	0.917
CW 15–19 years	-0.317 (-0.464 to -0.170)	**<0.001**

BR = Brazil; N = North; NE = Northeast; SE = Southeast; S = South; CW = Central-West; CKDMR = CKD mortality rates; HDI = human development index

Analyzing the 1 to 4 years range, there is no association between mortality rates in Brazil and its regions and the HDI.

In the age group 5 to 9 years, we observed a negative association with statistical significance in Brazil (HDI regression coefficient -0.051, 95% CI -0.077 to -0.025 *p* = 0.001) and in the SE region (HDI regression coefficient -0.092, 95% CI -0.126 to -0.059 *p*<0.001).

The age group 10 to 14 years shows negative correlation with statistical significance in Brazil (HDI regression coefficient -0.113, 95% CI -0.157 to -0.069 *p*<0.001), NE region (HDI regression coefficient -0.088, 95% CI -0.159 to -0.018 *p* = 0.024), SE region (HDI regression coefficient -0.165, 95% CI -0.227 to -0.103 *p*<0.001), and S region (HDI regression coefficient -0.104, 95% CI -0.173 to -0.036 *p* = 0.007).

The analysis of the 15 to 19 age group shows a negative association with statistical significance in Brazil (HDI regression coefficient -0.167, 95% CI -0.260 to -0.074 *p* = 0.002), N region (HDI regression coefficient -0.140, 95% CI -0.266 to -0.014 *p* = 0.042), SE region (HDI regression coefficient -0.250, 95% CI -0.388 to -0.112 *p* = 0.002), region S (HDI regression coefficient -0.132, 95% CI -0.247 to -0.017 *p* = 0.036) and region CW (regression coefficient -0.317, 95% CI -0.464 to -0.170 *p*<0.001).

The chart for CKDMR time trend adjusted by HDI for Brazil and its 5 age groups are shown in Fig 1B.

### 3.4. Regional correlation of CKDMR and HDI in different countries at two time periods in two different years (2000 and 2016)

**Fig 2:**
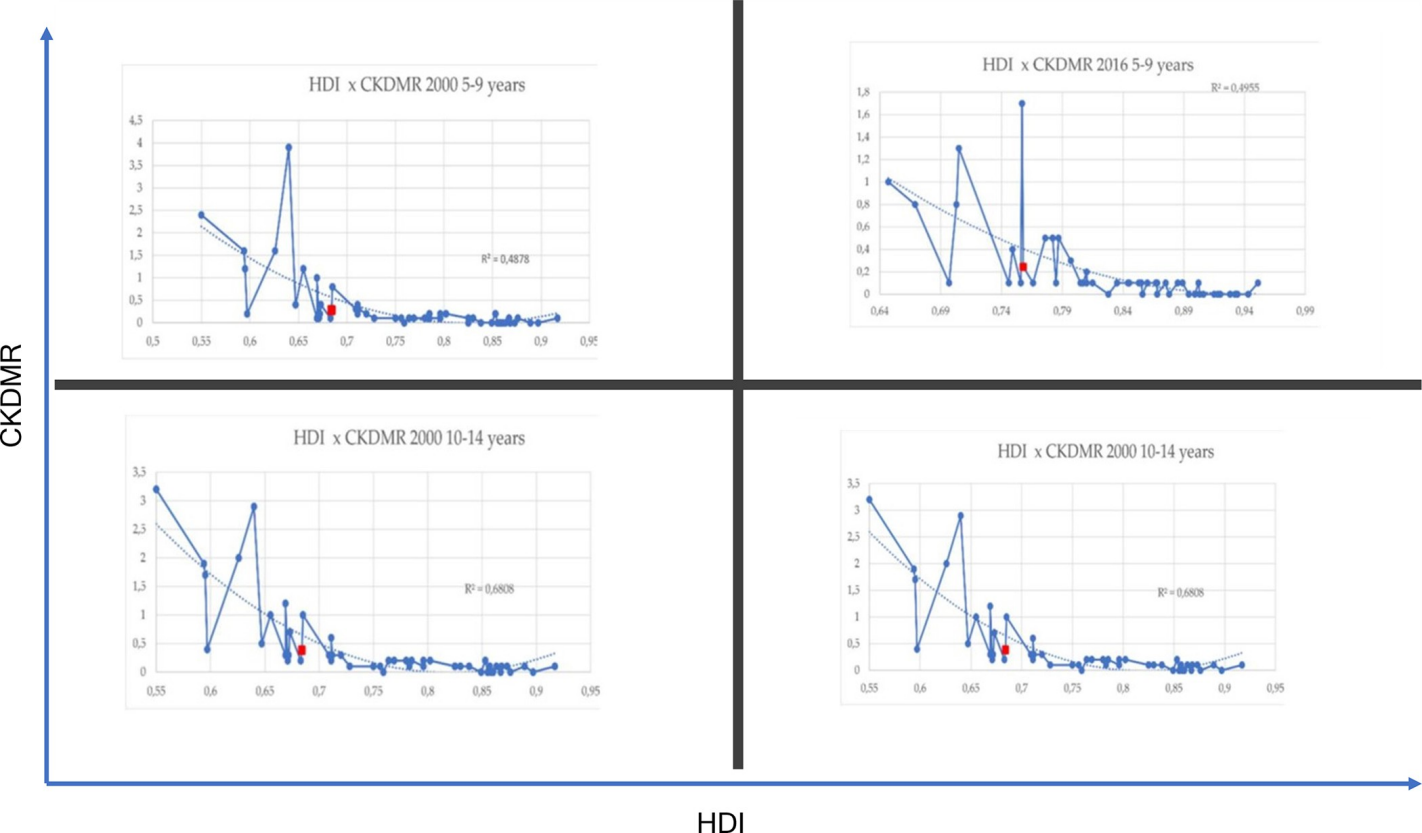
Comparative CKDMR x HDI in Brazil and WHO European Region for ages 5–9 and 10–14 years old in 2000 and 2016. CKDMR = CKD mortality rate; HDI = human development index Brazil.

We analyzed the correlation of HDI and CKDMR with the data contained in the supplementary material of the article “Causes of death among children aged 5 to 14 years in the WHO European Region: a systematic analysis for the Global Burden of Disease Study 2016” [11] with the authors’ permission. We used annual HDI values in the years 2000 and 2016 and the number of deaths from CKD in Europe and its various countries listed in the study, and in Brazil. The results are presented in Fig 2 with the curve estimated with a quadratic model, indicating an inverse relationship between HDI and CKD mortality in the countries studied.

## 4. Discussion

A demographic and epidemiological transition observed in low- and middle-income countries has produced a growing burden of non-communicable diseases and a decline in infectious diseases, associated with increasing urbanization, inadequate sanitation, suboptimal infection control and reproductive health, environmental hazards, and changes in lifestyle and diet observed in these populations [6]. The World Bank’s data qualifies Brazil as an upper-middle-income country [23].

A public health care system was implemented in Brazil in the year 1990 named Brazilian Unified Health System (*SUS—Sistema Único de Saúde*), with the principles of universality, integrality, equity, decentralization, and social participation [24]. In the 2000s, Brazil observed a combination of social and economic policies with growth in income and education, associated with regional developmental strategies. It resulted in a reduction in poverty and inequalities associated with a better distribution of income in the population, and a slowing in population growth because of lower fertility [25]. Unfortunately, as observed in low- and middle-income countries, it often exhibits a lack of evidence-based policy and care provisions, shortage and maldistribution of care providers, absence of organized primary care in many counties, fragmentation, and disparities in access and quality, and reliance on unproven therapies [26].

Between 1990 and 2015, Brazil made significant progress in reducing the mortality rate among children under the age of 5, achieving a reduction of 67.6%. During this period, there was a shift in the leading cause of death for this age group, with congenital anomalies becoming the second most prominent cause by 2015 [27].

Among these congenital anomalies, one of particular concern is the occurrence of kidney and urinary tract anomalies, collectively known as congenital anomalies of the kidney and urinary tract (CAKUT). This condition has emerged as a significant contributor to chronic kidney disease (CKD) in children, highlighting its importance in healthcare [28].

CKD is a noncommunicable disease recognized as a major public health issue worldwide, representing the twelfth leading cause of death in 2017 [29], with the fastest growth occurring in low-income and middle-income countries. A reduced life expectancy and an impaired quality of life are observed because of the higher risk observed for cardiovascular disease and infections [1].

The CKD in pediatrics is associated with genetic background predisposition, low birth weight, inadequate diets, infectious diseases, and exposures to toxins, with an incidence and prevalence rising strongly associated with lower socioeconomic status and ethnic groups [30].

Kidney failure is the most severe form of CKD, and it often needs the use of kidney replacement therapy (KRT), including dialysis and kidney transplantation. In Brazil, the KRT procedures are classified as complex, involving high technology and excessive cost. They are offered by SUS through the public administration and private companies with or without profit, called complementary networks. The public administration accounts for 13%, while the private companies with profit account for 62% of the KRT [31].

Almost 65% of Brazil’s dialysis units are located in the S and SE regions [13]. These KRT procedures were contracted with health services according to the provider’s offer, and their access has always occurred through spontaneous demand, not through regulation. It makes the distribution of health services and equipment unequal, creating regional inequalities, which haven’t been solved by SUS until these days [32]. The increase in the supply of KRT treatments in Brazil by the private system under the regulation of SUS allowed greater access for the population. There are few studies about mortality in children and adolescents associated with CKD in Brazil and around the world and the impact of HDI on the mortality of these populations.

Our study demonstrated a positive correlation between the mortality in children less than 1 year old and HDI in all regions of Brazil, probably related to the greater diagnosis of CKD in this age group by the improvement in health care and awareness of CKD, associated with the difficulties related to the management and treatment of this age group. This improvement has also led to a negative correlation between the mortality in ages 5 to 9 years old and HDI in Brazil and SE region, a negative correlation in Brazil and NE, S and SE regions for the 10 to 14 years old group, and in Brazil, N, S, SE, and CW regions for the 15 to 19 years old group. We observed stability in the mortality of children between 1 and 4 years of age. It could be the result of an improvement in the diagnosis of CKD and a real increase of cases due to epidemiological transition as hypothesized for the increase of mortality in the age group under 1 year, associated with a probable improvement in the treatment of CKD as proposed for the decreasing of mortality in the age groups over 5 years of age. The demographic and epidemiologic transition occurred more quickly in the SE, CW, and S regions, while in the N and NE, we are still able to see signs of our regional inequality. The mortality rates are already stabilized in the SE, while a significant drop still occurs in the NE and N. This could be explained by the hypothesis that the epidemiological transitions are still in the course in NE and N, while this transition is probably close to being consolidated in SE, CW, and S regions. Another reason could be the crescent number of patients with CKD diagnosis and larger access to the management and treatment available for this disease in the more developed regions of Brazil, resulting in a decline in mortality rates.

We used the correlation of HDI and CDKMR between European countries, without spatial analysis, to demonstrate that this association was also significant within the same period in countries with different HDIs. We do that because someone could ask if the temporal correlation between HDI and CDK observed in Brazil could be explained by the fact that the Brazilian HDI increased over the years and be solely due to the temporal trend without the need for predictors.

The results obtained in our study compared with European data [11] confirm the correlation between the increase in the HDI and the decrease in the mortality rates in these countries. Mortality rates and HDI in 2000 and 2016 for Brazil and Europe are presented in S2 and S3 Tables.

One notable aspect of the paper is its descriptive study design, which is particularly suited for generating hypotheses to be further explored in subsequent analytical designs such as cohort or case-control studies. This design choice emphasizes the initial exploration of trends and patterns, laying the foundation for more in-depth investigations into causal relationships and risk factors.

Our study is an ecological study that allows us to make some causal inferences in the regions studied, not allowing extrapolations for analysis of individuals because of the heterogeneity of the distribution and exposure to the risk factors and confounders within the population of each region (ecological fallacy) [33].

The data obtained from the SIM of DATASUS relies on the quality of completion of death certificates, the causal linkage between the reported ICD-10 and the mortality event, incorrectly generated and entered data in the system, lack of data, and the absence of individual patient information. The SIM of Brazil has been evaluated by WHO as an intermediate quality system [34].

As we know HDI is a proxy variable in our study and some hypotheses can be raised to be investigated in further analytical studies to explain its correlation with CKDMR estimated.

The HDI does not include indicators of inequalities within countries or regions, such as access to healthcare or the quality of services provided to the population, which limits the relevant socioeconomic variables for studying mortality in pediatric CKD. For example, in recent years, there has been a substantial increase in the number of graduated physicians and an increase in regulated medical courses in Brazil. Unfortunately, Brazil has an irregular distribution, with predominance in the capitals and the Southeast region. Few Brazilian states have a homogeneous distribution of general practitioners or specialists between the capital and inner cities.

CKD in childhood presents with often nonspecific signs and symptoms such as anemia, hypertension, and growth retardation. As a result, several children with CKD die without a diagnosis. The cases diagnosed with CKD end up being transferred to the large centers, mainly in the SE region, which promotes the migration of the child and family members for care and treatment in this region, being registered as belonging to the region to which they migrated and not the original region [35].

## 5. Conclusions

Our study confirmed the correlation between the increase in the HDI and the decrease in the mortality rates. The CKDMR is still unacceptably high in Brazil. It is imperative to reduce the CKD mortality rate in children and adolescents in our country, especially in less populated regions with improved access to specialized health services, through a set of public policies: the improvement of programs for prevention and diagnosis of CKD in primary care level, assessment for the management of risk factors to decrease disease progression and treatment for the causes and complications of CKD and increasing access to KRT. These measures can improve the treatment of one of Brazil’s important causes of non-communicable diseases. Further studies are essential to establish management strategies and approach to children and adolescents with CKD, reducing mortality rates in this population.

## Supporting information

S1 Data CKDMR in Brazil. (XLSX)

S1 Table Estimated annual percent changes of CKD mortality rates from the segmented regression annual percentage changes analyses (sAPC), the conventional annual percentage changes analysis (cAPC), and average annual percentage changes analysis (AAPC) with 95 percent confidence intervals: 1996–2017. Table legend: BR = Brazil; N = North; NE = Northeast; SE = Southeast; S = South; CW = Central-West.
(DOCX)

S2 Table CKDMR x HDI in WHO European Region and Brazil for children 5–9 years old. (DOCX)

S3 Table CKDMR x HDI in WHO European Region and Brazil for children 10–14 years old. (DOCX)

S1 Text Statistical analysis. (DOCX)
