# Supplementary material for: Pediatric chronic kidney disease mortality in Brazil—A time trend analysis
Source: PLOS Glob Public Health. 2024 Jan 24;4(1):e0002304. doi: 10.1371/journal.pgph.0002304 (PMC10807842; doi:10.1371/journal.pgph.0002304)
Supplement: S1 Table — Table legend: BR = Brazil; N = North; NE = Northeast; SE = Southeast; S = South; CW = Central-West. (DOCX) [file pgph.0002304.s002.docx]

| **Supplementary Table 1. Estimated annual percent changes of CKD mortality rates from the segmented regression annual percentage changes analyses (sAPC), the conventional annual percentage changes analysis (cAPC), and average annual percentage changes analysis (AAPC) with 95 percent confidence intervals: 1996–2017.** | | | | | | | | | | | | |
| --- | --- | --- | --- | --- | --- | --- | --- | --- | --- | --- | --- | --- |
|  | Segment regression analysis (1996-2017) | | | | | | | | | | | |
|  | Trend 1 | | Trend 2 | | Trend 3 | | | | 1996-2017 | | | |
|  | Years | sAPC (percent) | Years | sAPC (percent) | | Years | sAPC (percent) | cAPC (percent) | | 95% CI | AAPC (percent) | 95% CI |
| **BR <1y** | | | | | | | | | | | | |
| All sexes | 1996-2001 | -2.5 (-6.9;2.1) | 2001-2017 | 4.1* (3.4;4.9) | |  |  | 3.2* | | 2.5;3.8 | 2.5* | 1.4;3.7 |
| M | 1996-2001 | -2.3 (-8.8;4.5) | 2001-2017 | 4.4* (3.4;5.5) | |  |  | 3.4* | | 2.6;4.2 | 2.8* | 1.1;4.5 |
| F | 1996-2017 | 2.5* (1.4;3.5) |  |  | |  |  | 2.5* | | 1.4;3.5 | 2.5* | 1.4;3.5 |
| **BR 1–4y** | | | | | | | | | | | | |
| All sexes | 1996-2017 | 0.5 (-0.3;1.2) |  |  | |  |  | 0.5 | | -0.3;1.2 | 0.5 | -0.3;1.2 |
| M | 1996-2017 | 0.6 (-0.7;1.9) |  |  | |  |  | 0.6 | | -0.7;1.9 | 0.6 | -0.7;1.9 |
| F | 1996-2017 | 0.2 (-1.5;1.9) |  |  | |  |  | 0.2 | | -1.5;1.9 | 0.2 | -1.5;1.9 |
| **BR 5–9y** | | | | | | | | | | | | |
| All sexes | 1996-2017 | -1.6* (-2.6; -0.6) |  |  | |  |  | -1.6* | | -2.6; -0.6 | -1.6* | -2.6; -0.6 |
| M | 1996-2017 | -1.1 (-2.3;0.1) |  |  | |  |  | -1.1 | | -2.3;0.1 | -1.1 | -2.3;0.1 |
| F | 1996-2017 | -1.8* (-3.5;0.0) |  |  | |  |  | -1.8* | | -3.5;0.0 | -1.8* | -3.5;0.0 |
| **BR 10–14y** | | | | | | | | | | | | |
| All sexes | 1996-2001 | -10.3* (-16.2; -3.9) | 2001-2017 | -1.0 (-2.4;0.5) | |  |  | -2.8* | | -3.9; -1.7 | -3.3* | -5.0; -1.5 |
| M | 1996-2017 | -2.0* (-3.6; -0.4) |  |  | |  |  | -2.0* | | -3.6; -0.4 | -2.0* | -3.6; -0.4 |
| F | 1996-2017 | -3.4* (-4.6; -2.2) |  |  | |  |  | -3.4* | | -4.6; -2.2 | -3.4* | -4.6; -2.2 |
| **BR 15–19y** | | | | | | | | | | | | |
| All sexes | 1996-2003 | -7.0* (--10.7; -3.2) | 2003-2017 | 0.5 (-1.1;2.1) | |  |  | -1.8* | | -2.8; -0.7 | -2.1* | -3.6; -0.5 |
| M | 1996-2002 | -9.6* (-14.9; -3.9) | 2002-2017 | 1.3 (-0.4;3.0) | |  |  | -1.3* | | -2.6; -0.1 | -1.9 | -3.8;0.0 |
| F | 1996-2017 | -2.1* (-3.1; -1.0) |  |  | |  |  | -2.1* | | -3.1; -1.0 | -2.1* | -3.1; -1.0 |
| **CW <1y** | | | | | | | | | | | | |
| All sexes | 1996-2017 | 2.8* (0.6; 5.1) |  |  | |  |  | 2.8* | | 0.6; 5.1 | 2.8* | 0.6; 5.1 |
| M | 1996-2017 | 1.9 (-0.8;4.7) |  |  | |  |  | 1.9 | | -0.8;4.7 | 1.9 | -0.8;4.7 |
| F | 1996-2008 | 6.6 (-1.7;15.6) | 2008-2011 | -60.0 (-87.2;25.4) | | 2011-2017 | 8.5 (-12.6;34.8) | -12.8* | | -17.8; -7.5 | -6.8 | -21.0;9.9 |
| **CW 1–4y** | | | | | | | | | | | | |
| All sexes | 1996-2017 | 2.5* (0.6;4.4) |  |  | |  |  | 2.5* | | 0.6;4.4 | 2.5* | 0.6;4.4 |
| M | 1996-2017 | 1.5 (-1.2;4.3) |  |  | |  |  | 1.5 | | -1.2;4.3 | 1.5 | -1.2;4.3 |
| F | 1996-2017 | 4.4* (1.4;7.4) |  |  | |  |  | 4.4* | | 1.4;7.4 | 4.4* | 1.4;7.4 |
| **CW 5–9y** | | | | | | | | | | | | |
| All sexes | 1996-2017 | -0.4 (-3.5;2.8) |  |  | |  |  | -0.4 | | -3.5;2.8 | -0.4 | -3.5;2.8 |
| M | 1996-2017 | 2.1 (-1.5;6.0) |  |  | |  |  | 2.1 | | -1.5;6.0 | 2.1 | -1.5;6.0 |
| F | 1996-2017 | -0.8 (-5.6;4.2) |  |  | |  |  | -0.8 | | -5.6;4.2 | -0.8 | -5.6;4.2 |
| **CW 10–14y** | | | | | | | | | | | | |
| All sexes | 1996-2017 | -0.7 (-3.4;2.1) |  |  | |  |  | -0.7 | | -3.4;2.1 | -0.7 | -3.4;2.1 |
| M | 1996-2017 | -1.2 (-5.6;3.4) |  |  | |  |  | -1.2 | | -5.6;3.4 | -1.2 | -5.6;3.4 |
| F | 1996-2017 | -0.3 (-3.1;2.5) |  |  | |  |  | -0.3 | | -3.1;2.5 | -0.3 | -3.1;2.5 |
| **CW 15–19y** | | | | | | | | | | | | |
| All sexes | 1996-2017 | -3.0* (-4.4; -1.5) |  |  | |  |  | -3.0* | | -4.4; -1.5 | -3.0* | -4.4; -1.5 |
| M | 1996-2017 | -1.0 (-4.0;2.1) |  |  | |  |  | -1.0 | | -4.0;2.1 | -1.0 | -4.0;2.1 |
| F | 1996-2017 | -4.1* (-6.3; -1.9) |  |  | |  |  | -4.1* | | -6.3; -1.9 | -4.1* | -6.3; -1.9 |
| **N <1y** | | | | | | | | | | | | |
| All sexes | 1996-2017 | 4.0* (1.7;6.3) |  |  | |  |  | 4.0* | | 1.7;6.3 | 4.0* | 1.7;6.3 |
| M | 1996-2017 | 4.3* (1.3;7.4) |  |  | |  |  | 4.3* | | 1.3;7.4 | 4.3* | 1.3;7.4 |
| F | 1996-2017 | 2.5 (-0.8;5.9) |  |  | |  |  | 2.5 | | -0.8;5.9 | 2.5 | -0.8;5.9 |
| **N 1–4y** | | | | | | | | | | | | |
| All sexes | 1996-2017 | 0.4 (-2.4;3.2) |  |  | |  |  | 0.4 | | -2.4;3.2 | 0.4 | -2.4;3.2 |
| M | 1996-2017 | 1.9 (-2.0;5.9) |  |  | |  |  | 1.9 | | -2.0;5.9 | 1.9 | -2.0;5.9 |
| F | 1996-2017 | -0.9 (-4.3;2.6) |  |  | |  |  | -0.9 | | -4.3;2.6 | -0.9 | -4.3;2.6 |
| **N 5–9y** | | | | | | | | | | | | |
| All sexes | 1996-2017 | -1.6 (-4.7;1.5) |  |  | |  |  | -1.6 | | -4.7;1.5 | -1.6 | -4.7;1.5 |
| M | 1996-2017 | -2.7 (-6.3;1.0) |  |  | |  |  | -2.7 | | -6.3;1.0 | -2.7 | -6.3;1.0 |
| F | 1996-2017 | -0.4 (-3.7;3.0) |  |  | |  |  | -0.4 | | -3.7;3.0 | -0.4 | -3.7;3.0 |
| **N 10–14y** | | | | | | | | | | | | |
| All sexes | 1996-2017 | -1.2 (-3.6;1.2) |  |  | |  |  | -1.2 | | -3.6;1.2 | -1.2 | -3.6;1.2 |
| M | 1996-2017 | -1.2 (-4.9;2.6) |  |  | |  |  | -1.2 | | -4.9;2.6 | -1.2 | -4.9;2.6 |
| F | 1996-2017 | -0.5 (-3.4;2.5) |  |  | |  |  | -0.5 | | -3.4;2.5 | -0.5 | -3.4;2.5 |
| **N 15–19y** | | | | | | | | | | | | |
| All sexes | 1996-2017 | -1.4* (-2.9; -0.0) |  |  | |  |  | -1.4* | | -2.9; -0.0 | -1.4* | -2.9; -0.0 |
| M | 1996-2017 | -0.7 (-3.6;2.4) |  |  | |  |  | -0.7 | | -3.6;2.4 | -0.7 | -3.6;2.4 |
| F | 1996-2017 | -1.6 (-3.6;0.4) |  |  | |  |  | -1.6 | | -3.6;0.4 | -1.6 | -3.6;0.4 |
| **NE <1y** | | | | | | | | | | | | |
| All sexes | 1996-2017 | 4.3* (2.9;5.7) |  |  | |  |  | 4.3* | | 2.9;5.7 | 4.3* | 2.9;5.7 |
| M | 1996-2017 | 5.3* (3.2;7.3) |  |  | |  |  | 5.3* | | 3.2;7.3 | 5.3* | 3.2;7.3 |
| F | 1996-2017 | 2.8* (0.9;4.8) |  |  | |  |  | 2.8* | | 0.9;4.8 | 2.8* | 0.9;4.8 |
| **NE 1–4y** | | | | | | | | | | | | |
| All sexes | 1996-2017 | 1.0 (-0.6;2.6) |  |  | |  |  | 1.0 | | -0.6;2.6 | 1.0 | -0.6;2.6 |
| M | 1996-2017 | 1.6 (-0.1;3.3) |  |  | |  |  | 1.6 | | -0.1;3.3 | 1.6 | -0.1;3.3 |
| F | 1996-2017 | 0.0 (-3.4;3.6) |  |  | |  |  | 0.0 | | -3.4;3.6 | 0.0 | -3.4;3.6 |
| **NE 5–9y** | | | | | | | | | | | | |
| All sexes | 1996-2017 | -0.1 (-1.9;1.8) |  |  | |  |  | -0.1 | | -1.9;1.8 | -0.1 | -1.9;1.8 |
| M | 1996-2017 | -0.2 (-2.5;2.2) |  |  | |  |  | -0.2 | | -2.5;2.2 | -0.2 | -2.5;2.2 |
| F | 1996-2017 | 0.4 (-2.1;3.0) |  |  | |  |  | 0.4 | | -2.1;3.0 | 0.4 | -2.1;3.0 |
| **NE 10–14y** | | | | | | | | | | | | |
| All sexes | 1996-2017 | -1.8* (-3.3; -0.3) |  |  | |  |  | -1.8* | | -3.3; -0.3 | -1.8* | -3.3; -0.3 |
| M | 1996-2017 | -1.3 (-3.0;0.5) |  |  | |  |  | -1.3 | | -3.0;0.5 | -1.3 | -3.0;0.5 |
| F | 1996-2017 | -2.2* (-4.1; -0.2) |  |  | |  |  | -2.2* | | -4.1; -0.2 | -2.2* | -4.1; -0.2 |
| **NE 15–19y** | | | | | | | | | | | | |
| All sexes | 1996-2017 | -0.4 (-1.6;0.8) |  |  | |  |  | -0.4 | | -1.6;0.8 | -0.4 | -1.6;0.8 |
| M | 1996-2017 | 0.9 (-0.8;2.6) |  |  | |  |  | 0.9 | | -0.8;2.6 | 0.9 | -0.8;2.6 |
| F | 1996-2017 | -1.3 (-2.7;0.1) |  |  | |  |  | -1.3 | | -2.7;0.1 | -1.3 | -2.7;0.1 |
| **S <1y** | | | | | | | | | | | | |
| All sexes | 1996-2017 | 3.1* (1.2;5.1) |  |  | |  |  | 3.1* | | 1.2;5.1 | 3.1* | 1.2;5.1 |
| M | 1996-2017 | 2.9* (1.1;4.9) |  |  | |  |  | 2.9* | | 1.1;4.9 | 2.9* | 1.1;4.9 |
| F | 1996-2017 | 2.7 (-0.4;5.9) |  |  | |  |  | 2.7 | | -0.4;5.9 | 2.7 | -0.4;5.9 |
| **S 1–4y** | | | | | | | | | | | | |
| All sexes | 1996-2017 | 1.0 (-2.2;4.4) |  |  | |  |  | 1.0 | | -2.2;4.4 | 1.0 | -2.2;4.4 |
| M | 1996-2017 | 0.1 (-3.9;4.4) |  |  | |  |  | 0.1 | | -3.9;4.4 | 0.1 | -3.9;4.4 |
| F | 1996-2017 | 1.7 (-3.0;6.7) |  |  | |  |  | 1.7 | | -3.0;6.7 | 1.7 | -3.0;6.7 |
| **S 5–9y** | | | | | | | | | | | | |
| All sexes | 1996-2017 | -1.3 (-4.7;2.2) |  |  | |  |  | -1.3 | | -4.7;2.2 | -1.3 | -4.7;2.2 |
| M | 1996-2017 | 0.9 (-3.5;5.5) |  |  | |  |  | 0.9 | | -3.5;5.5 | 0.9 | -3.5;5.5 |
| F | 1996-2017 | -2.8 (-6.5;1.0) |  |  | |  |  | -2.8 | | -6.5;1.0 | -2.8 | -6.5;1.0 |
| **S 10–14y** | | | | | | | | | | | | |
| All sexes | 1996-2017 | -2.8* (-5.0; -0.4) |  |  | |  |  | -2.8* | | -5.0; -0.4 | -2.8* | -5.0; -0.4 |
| M | 1996-2017 | 1.1 (-3.0;5.3) |  |  | |  |  | 1.1 | | -3.0;5.3 | 1.1 | -3.0;5.3 |
| F | 1996-2017 | -4.9*(-7.6; -2.1) |  |  | |  |  | -4.9* | | -7.6; -2.1 | -4.9* | -7.6; -2.1 |
| **S 15–19y** | | | | | | | | | | | | |
| All sexes | 1996-2017 | -1.6* (-3.2; -0.0) |  |  | |  |  | -1.6* | | -3.2; -0.0 | -1.6* | -3.2; -0.0 |
| M | 1996-2017 | -3.3* (-5.6; -1.1) |  |  | |  |  | -3.3* | | -5.6; -1.1 | -3.3* | -5.6; -1.1 |
| F | 1996-2017 | -0.8 (-3.2;1.6) |  |  | |  |  | -0.8 | | -3.2;1.6 | -0.8 | -3.2;1.6 |
| **SE <1y** | | | | | | | | | | | | |
| All sexes | 1996-2002 | -5.4 (-11.3;0.8) | 2002-2017 | 4.7* (3.3;6.2) | |  |  | 2.6* | | 1.4;3.8 | 1.7 | -0.2;3.7 |
| M | 1996-2001 | -8.8 (-19.1;2.7) | 2001-2017 | 5.3* (3.4;7.3) | |  |  | 3.0* | | 1.5;4.5 | 1.8 | -1.2;4.8 |
| F | 1996-2017 | 1.9* (0.2;3.6) |  |  | |  |  | 1.9* | | 0.2;3.6 | 1.9* | 0.2;3.6 |
| **SE 1–4y** | | | | | | | | | | | | |
| All sexes | 1996-2017 | -0.1 (-1.5;1.3) |  |  | |  |  | -0.1 | | -1.5;1.3 | -0.1 | -1.5;1.3 |
| M | 1996-2017 | -0.4 (-2.4;1.6) |  |  | |  |  | -0.4 | | -2.4;1.6 | -0.4 | -2.4;1.6 |
| F | 1996-2017 | -0.2 (-2.4;2.1) |  |  | |  |  | -0.2 | | -2.4;2.1 | -0.2 | -2.4;2.1 |
| **SE 5–9y** | | | | | | | | | | | | |
| All sexes | 1996-2017 | -3.7* (-5.1; -2.2) |  |  | |  |  | -3.7* | | -5.1; -2.2 | -3.7* | -5.1; -2.2 |
| M | 1996-2017 | -1.9 (-4.1;0.4) |  |  | |  |  | -1.9 | | -4.1;0.4 | -1.9 | -4.1;0.4 |
| F | 1996-2017 | -4.7* (-7.0; -2.5) |  |  | |  |  | -4.7* | | -7.0; -2.5 | -4.7* | -7.0; -2.5 |
| **SE 10–14y** | | | | | | | | | | | | |
| All sexes | 1996-2003 | -13.7* (-20.0; -6.8) | 2003-2007 | 9.8 (-19.5;49.8) | | 2007-2017 | -6.1* (-11.3; -0.5) | -4.9* | | -6.6; -3.1 | -5.9 | -11.8;0.3 |
| M | 1996-2017 | -3.2* (-5.6; -0.7) |  |  | |  |  | -3.2* | | -5.6; -0.7 | -3.2* | -5.6; -0.7 |
| F | 1996-2017 | -6.0* (-7.9; -4.1) |  |  | |  |  | -6.0* | | -7.9; -4.1 | -6.0* | -7.9; -4.1 |
| **SE 15–19y** | | | | | | | | | | | | |
| All sexes | 1996-2003 | -11.4* (-15.5; -7.0) | 2003-2017 | 1.3 (-0.7; 3.3) | |  |  | -2.8* | | -4.3; -1.3 | -3.1* | -4.9; -1.2 |
| M | 1996-2002 | -13.6* (-21.5; -4.9) | 2002-2017 | 1.0 (-1.9;4.1) | |  |  | -2.9* | | -4.7; -1.1 | -3.4* | -6.4; -0.2 |
| F | 1996-2003 | -10.9* (-16.3; -5.2) | 2003-2017 | 1.3 (-1.4;4.0) | |  |  | -2.7* | | -4.3; -1.0 | -3.0* | -5.4; -0.5 |
| BR=Brazil; N=North; NE=Northeast; SE=Southeast; S=South; CW=Central-West. | | | | | | | | | | | | |
