# Supplementary material for: Pediatric chronic kidney disease mortality in Brazil—A time trend analysis
Source: PLOS Glob Public Health. 2024 Jan 24;4(1):e0002304. doi: 10.1371/journal.pgph.0002304 (PMC10807842; doi:10.1371/journal.pgph.0002304)
Supplement: S2 Table — (DOCX) [file pgph.0002304.s003.docx]

| **Supplementary Table 2 – CKDMR x HDI in WHO European Region and Brazil for children 5–9 years old** | | | | |
| --- | --- | --- | --- | --- |
| **Country** | **2000 HDI** | **2000 CKDMR** | **2016 HDI*** | **2016 CKDMR** |
| Norway | 0.917 | 0.1 | 0.951 | 0.1 |
| Switzerland | 0.889 | 0 | 0.943 | 0 |
| Germany | 0.868 | 0 | 0.934 | 0 |
| Ireland | 0.857 | 0 | 0.934 | 0 |
| Iceland | 0.86 | 0 | 0.933 | 0 |
| Sweden | 0.897 | 0 | 0.932 | 0 |
| Denmark | 0.863 | 0 | 0.928 | 0 |
| Netherlands | 0.876 | 0.1 | 0.928 | 0 |
| UK | 0.867 | 0.1 | 0.92 | 0 |
| Finland | 0.858 | 0 | 0.918 | 0 |
| Belgium | 0.873 | 0 | 0.915 | 0 |
| Austria | 0.838 | 0 | 0.906 | 0 |
| Luxembourg | 0.855 | 0 | 0.903 | 0 |
| Israel | 0.853 | 0.2 | 0.902 | 0.1 |
| France | 0.849 | 0 | 0.899 | 0 |
| Slovenia | 0.825 | 0 | 0.894 | 0 |
| Spain | 0.825 | 0.1 | 0.889 | 0.1 |
| Czech republic | 0.796 | 0.2 | 0.885 | 0.1 |
| Italy | 0.83 | 0.1 | 0.878 | 0 |
| Malta | 0.783 | 0.1 | 0.875 | 0.1 |
| Estonia | 0.78 | 0.1 | 0.868 | 0 |
| Greece | 0.796 | 0.1 | 0.868 | 0.1 |
| Cyprus | 0.802 | 0.2 | 0.867 | 0.1 |
| Poland | 0.785 | 0.1 | 0.86 | 0.1 |
| Andorra | 0.759 | 0 | 0.856 | 0 |
| Lithuania | 0.756 | 0.1 | 0.855 | 0.1 |
| Slovakia | 0.764 | 0.1 | 0.853 | 0.1 |
| Portugal | 0.785 | 0.2 | 0.845 | 0.1 |
| Latvia | 0.728 | 0.1 | 0.844 | 0.1 |
| Hungary | 0.769 | 0.1 | 0.835 | 0.1 |
| Croatia | 0.75 | 0.1 | 0.828 | 0 |
| Russia | 0.72 | 0.2 | 0.815 | 0.1 |
| Bulgaria | 0.712 | 0.3 | 0.81 | 0.2 |
| Montenegro | 0.711 | 0.4 | 0.81 | 0.1 |
| Romania | 0.709 | 0.3 | 0.807 | 0.1 |
| Belarus | 0.683 | 0.1 | 0.805 | 0.1 |
| Kazakhstan | 0.685 | 0.8 | 0.797 | 0.3 |
| Türkiye | 0.655 | 1.2 | 0.787 | 0.5 |
| Serbia | 0.711 | 0.2 | 0.785 | 0.1 |
| Albania | 0.669 | 1 | 0.782 | 0.5 |
| Georgia | 0.673 | 0.4 | 0.776 | 0.5 |
| Bosnia and Herzegovina | 0.672 | 0.2 | 0.766 | 0.1 |
| **Brazil** | **0.684** | **0.28** | **0.758** | **0.24** |
| Azerbaijan | 0.64 | 3.9 | 0.757 | 1.7 |
| Macedonia | 0.669 | 0.1 | 0.756 | 0.1 |
| Armenia | 0.647 | 0.4 | 0.749 | 0.4 |
| Ukraine | 0.671 | 0.1 | 0.746 | 0.1 |
| Turkmenistan | 0.626 | 1.6 | 0.705 | 1.3 |
| Uzbekistan | 0.595 | 1.2 | 0.703 | 0.8 |
| Moldavia | 0.597 | 0.2 | 0.697 | 0.1 |
| Kyrgyzstan | 0.594 | 1.6 | 0.669 | 0.8 |
| Tajikistan | 0.55 | 2.4 | 0.647 | 1 |
| Sorted by HDI 2016* | | | | |
