# Supplementary material for: Pediatric chronic kidney disease mortality in Brazil—A time trend analysis
Source: PLOS Glob Public Health. 2024 Jan 24;4(1):e0002304. doi: 10.1371/journal.pgph.0002304 (PMC10807842; doi:10.1371/journal.pgph.0002304)
Supplement: S3 Table — (DOCX) [file pgph.0002304.s004.docx]

| **Supplementary Table 3 – CKDMR x HDI in WHO European Region and Brazil for children 10–14 years old** | | | | |
| --- | --- | --- | --- | --- |
| **Country** | **2000 HDI** | **2000 CKDMR** | **2016 HDI*** | **2016 CKDMR** |
| Norway | 0.917 | 0.1 | 0.95 | 0.00 |
| Switzerland | 0.889 | 0.1 | 0.94 | 0.00 |
| Germany | 0.868 | 0.1 | 0.93 | 0.00 |
| Ireland | 0.857 | 0.1 | 0.93 | 0.00 |
| Iceland | 0.86 | 0 | 0.93 | 0.00 |
| Sweden | 0.897 | 0 | 0.93 | 0.00 |
| Denmark | 0.863 | 0.1 | 0.93 | 0.10 |
| Netherlands | 0.876 | 0 | 0.93 | 0.00 |
| UK | 0.867 | 0 | 0.92 | 0.00 |
| Finland | 0.858 | 0 | 0.92 | 0.00 |
| Belgium | 0.873 | 0.1 | 0.92 | 0.00 |
| Austria | 0.838 | 0.1 | 0.91 | 0.10 |
| Luxembourg | 0.855 | 0 | 0.90 | 0.00 |
| Israel | 0.853 | 0.2 | 0.90 | 0.10 |
| France | 0.849 | 0 | 0.90 | 0.00 |
| Slovenia | 0.825 | 0.1 | 0.89 | 0.00 |
| Spain | 0.825 | 0.1 | 0.89 | 0.10 |
| Czech republic | 0.796 | 0.2 | 0.89 | 0.10 |
| Italy | 0.83 | 0.1 | 0.88 | 0.10 |
| Malta | 0.783 | 0.1 | 0.88 | 0.10 |
| Estonia | 0.78 | 0.2 | 0.87 | 0.10 |
| Greece | 0.796 | 0.1 | 0.87 | 0.10 |
| Cyprus | 0.802 | 0.2 | 0.87 | 0.10 |
| Poland | 0.785 | 0.2 | 0.86 | 0.10 |
| Andorra | 0.759 | 0 | 0.86 | 0.00 |
| Lithuania | 0.756 | 0.1 | 0.86 | 0.10 |
| Slovakia | 0.764 | 0.2 | 0.85 | 0.10 |
| Portugal | 0.785 | 0.2 | 0.85 | 0.10 |
| Latvia | 0.728 | 0.1 | 0.84 | 0.10 |
| Hungary | 0.769 | 0.2 | 0.84 | 0.20 |
| Croatia | 0.75 | 0.1 | 0.83 | 0.10 |
| Russia | 0.72 | 0.3 | 0.82 | 0.20 |
| Bulgaria | 0.712 | 0.3 | 0.81 | 0.30 |
| Montenegro | 0.711 | 0.6 | 0.81 | 0.30 |
| Romania | 0.709 | 0.3 | 0.81 | 0.20 |
| Belarus | 0.683 | 0.2 | 0.81 | 0.10 |
| Kazakhstan | 0.685 | 1 | 0.80 | 0.30 |
| Türkiye | 0.655 | 1 | 0.79 | 0.50 |
| Serbia | 0.711 | 0.2 | 0.79 | 0.10 |
| Albania | 0.669 | 1.2 | 0.78 | 0.90 |
| Georgia | 0.673 | 0.7 | 0.78 | 0.90 |
| Bosnia and Herzegovina | 0.672 | 0.3 | 0.77 | 0.20 |
| **Brazil** | **0.684** | **0.38** | **0.76** | **0.24** |
| Azerbaijan | 0.64 | 2.9 | 0.76 | 2.00 |
| Macedonia | 0.669 | 0.3 | 0.76 | 0.20 |
| Armenia | 0.647 | 0.5 | 0.75 | 0.60 |
| Ukraine | 0.671 | 0.2 | 0.75 | 0.10 |
| Turkmenistan | 0.626 | 2 | 0.71 | 1.80 |
| Uzbekistan | 0.595 | 1.7 | 0.70 | 1.50 |
| Moldavia | 0.597 | 0.4 | 0.70 | 0.30 |
| Kyrgyzstan | 0.594 | 1.9 | 0.67 | 1.00 |
| Tajikistan | 0.55 | 3.2 | 0.65 | 1.50 |
| Sorted by HDI 2016* | | | | |
