# Supplementary material for: Pediatric chronic kidney disease mortality in Brazil—A time trend analysis
Source: PLOS Glob Public Health. 2024 Jan 24;4(1):e0002304. doi: 10.1371/journal.pgph.0002304 (PMC10807842; doi:10.1371/journal.pgph.0002304)
Supplement: S1 Text — (DOCX) [file pgph.0002304.s005.docx]

Supplementary material: Statistical analysis

1. Time trend analysis

CKDMR was calculated for each age group (less than 1 year, 1 to 4, 5 to 9, 10 to 14, and 15 to 19) for Brazil as a whole and its different regions and expressed per 100,000 children per year. The APC and AAPC of each CKDMR were estimated using the Poisson variance in the corrected error option, while the presence of possible breaks in the temporal trends was assessed with the Joinpoint regression analysis. To identify changes in mortality rate trends, Joinpoint regression was estimated for every age and sex group by using the Joinpoint Regression Program, Version V4.8.0.1 (Statistical Research and Applications Branch, National Cancer Institute). In brief, by using mortality rates as inputs, this method identifies the year(s) when a trend change is produced, calculates the APC in rates between trend-change points, and it also estimates the AAPC in the whole period studied. When there are no join points (i.e., no changes in trend), APC is constant, so it equals the AAPC. Otherwise, the whole period is segmented by the points with a trend change. Then, AAPC is estimated as a weighted average of the estimated APC in each segment by using the segment lengths as weights. The number of join points is obtained using a permutation test via Monte Carlo resampling[19]. Once the number k of join points has been obtained, the different models with k join points are compared by estimating their Bayesian Information Criterion (BIC)[20].

To calculate the APC, the following model was used to estimate the CKDMR in each year:

$Ln\left( Yx \right)=b0+b1x$, where Ln (Yx) is the natural logarithm of the CKDMR in year x.

Then, the APC from year x to year x+1 is:

$$APC=\frac{exp(b0+b1\left( x+1 \right)-eb0+b1x)}{exp(b0+b1x)} x 100=(exp \left( b1 \right)-1) x100$$

Where *b*0 is the value of the intercept and *b*1 is the coefficient x determined by the joinpoint analysis from year x to year x+1.

And the AAPC is calculated as follows:

$$AAPC=\left( \frac{exp(s1*APC1+s2*APC2+\ldots+sn*APCn)}{exp(s1+s2+\ldots+sn)}-1 \right)x100$$

Where s1…sn is the number of the year of each segment between the joint point determined by the permutation test. APC1-APCn is the value of APC in each segment.

2. Time trend analysis for HDI regressor

The association between the annual HDI values in Brazil and the CKDMR in each age group in both sexes and its different regions in Brazil was assessed using the time series ARIMA models with regressor, which can be described by the following formula:

$$\text{Ŷ}\text{t}\text{-Θ}\text{1}\text{Y}\text{t-1}\text{=µ-Φ}\text{1}\text{e}\text{t-1}\text{+β(X}\text{t}\text{-Θ}\text{1}\text{X}\text{t-1}\text{)}$$

Where our HDI time trend analysis corresponds to X=HDI, β= HDI coefficient e=error, Y= estimated CSMR, Θ=slope coefficient, Φ= error coefficient[21].

3. Pairwise multiple comparison adjustment

To compare the proportion of death in the population by different regions in each age group, the chi-square test was used. To correct multiple comparisons, we use the Bonferroni procedure. As we made 75 comparisons, the new significant p-value cutoff is <0.0007.

4. Curve estimation to regional (countries) analysis (no spatial correlation) in the same period

To estimate the correlation between the HDI and CKDMR in the same periods (2000 and 2016) using the European Countries and Brazil, we use the CURVEFIT function of SPSS to determine the best curve model for our data. We have tried linear, logarithmic, inverse, quadratic, cubic, power, compound, and S-curve models. The quadratic model was selected because of the higher R2 (coefficient of determination). For our data, the quadratic curve model is described by the following formula:

Y = b0 + ($\text{β}$ 1 * x) + ($\text{β}$ 2 * x2).

Where our HDI regional analysis corresponds to X=HDI, βn= HDI coefficients, Y= estimated CKDMR[22].

Statistical analyzes were performed using the IBM SPSS Statistics 20 software and the Joinpoint regression program (V4.8.0.1, National Cancer).
